# Graphene Electro-Optical Switch Modulator by Adjusting Propagation Length Based on Hybrid Plasmonic Waveguide in Infrared Band

**DOI:** 10.3390/s20102864

**Published:** 2020-05-18

**Authors:** Ming Cai, Shulong Wang, Zhihong Liu, Yindi Wang, Tao Han, Hongxia Liu

**Affiliations:** Key Laboratory for Wide Band Gap Semiconductor Materials and Devices of Education, School of Microelectronics, Xidian University, Xi’an 710071, China; cm9999787@163.com (M.C.); zhliu@xidian.edu.cn (Z.L.); wyd4213@163.com (Y.W.); 15639119745@163.com (T.H.); hxliu@mail.xidian.edu.cn (H.L.)

**Keywords:** plasma modulator, graphene, SPP, propagation length, hybrid waveguide

## Abstract

A modulator is the core of many optoelectronic applications such as communication and sensing. However, a traditional modulator can hardly reach high modulation depth. In order to achieve the higher modulation depth, a graphene electro-optical switch modulator is proposed by adjusting propagation length in the near infrared band. The switch modulator is designed based on a hybrid plasmonic waveguide structure, which is comprised of an SiO_2_ substrate, graphene–Si–graphene heterostructure, Ag nanowire and SiO_2_ cladding. The propagation length of the hybrid plasmonic waveguide varies from 0.14 μm to 20.43 μm by the voltage tunability of graphene in 1550 nm incident light. A modulator with a length of 3 μm is designed based on the hybrid waveguide and it achieves about 100% modulation depth. The lower energy loss (~1.71 fJ/bit) and larger 3 dB bandwidth (~83.91 GHz) are attractive for its application in a photoelectric integration field. In addition, the excellent robustness (error of modulation effects lower than 8.84%) is practical in the fabrication process. Most importantly, by using the method of adjusting propagation length, other types of graphene modulators can also achieve about 100% modulation depth.

## 1. Introduction

Graphene, a two-dimensional material, was obtained by mechanical peeling in 2004 [1]. Due to the excellent optical and electrical properties, graphene has a great potential in the field of photodetectors [2,3], waveguides [4,5], optical modulators [6,7], supercapacitors [8,9], sensors [10,11] and so on. Scientists have found that the absorption rate of graphene with intrinsic monolayer thickness is about 2.3%, which is 50 times higher than gallium arsenide with same thickness and can be easily tuned by changing graphene conductivity in different gating voltage [12,13]. The mainly reason is that strong light–matter interaction in graphene can be controlled effectively by tuning the Fermi level (E_F_) with electrostatic gating. Based on this excellent tunability of the absorption property [14] through electrostatic gating, graphene can be used for designing electro-optical devices and has been widely applied in electro-optical modulators [15,16,17,18,19,20]. Graphene modulators have shown relative high modulation depth in different structures [19,20].

To provide subwavelength confinement and realize light manipulation on nanoscale devices, plasmonic waveguides based on surface plasmon polarizations (SPPs) wave have been researched a lot [21,22,23,24]. To further reduce the high optical loss and increase propagation length, hybrid plasmonic waveguides, constructed by surface plasmon waveguides and silicon waveguides [25], have been proposed. Since then, many kinds of hybrid plasmon waveguide structure have been researched to further enhance the waveguide performance [26,27,28,29]. However, there are only few works concerned with the modulation properties based on a hybrid plasmonic waveguide and the lack ways to achieve deeper modulation performance. For the excellent tunability of graphene conductivity, devices based on graphene have a potential in the subwavelength waveguides [30] and modulators [31,32,33]. The modulation depth can be achieved at 0.03 dB/um [34] and 0.6 dB/μm [35] for different graphene hybrid plasmonic waveguide modulators. 

Although there are many studies of the graphene-based modulator in recent years, there is no solution to obtain high modulation performance by the traditional method. In addition, for the better application in actual engineering, the higher bandwidth and lower energy consumption of modulators are also needed. In order to solve these problems, a novel graphene electro-optical switch modulator based on silver nanowire (GESMBOSN), by adjusting propagation length with a hybrid plasmonic waveguide, is proposed in the near infrared band (~1550 nm). It is worth mentioning that the tunability of optical propagation length in graphene waveguides has been ignored in the past research of graphene modulators. Using tunablity of propagation length to design graphene switch modulators, the proposed switch modulator successfully achieves about 100% modulation depth. The modulator also realizes lower energy loss and larger 3dB bandwidth in the near infrared band. Besides, the excellent robustness provides more convenient conditions for actual production. Most importantly, as a new method to design graphene switch modulators, other different structure’s graphene switch modulators can also apply this new method to achieve ~100% modulation depth.

## 2. Materials and Methods

**Figure 1:**
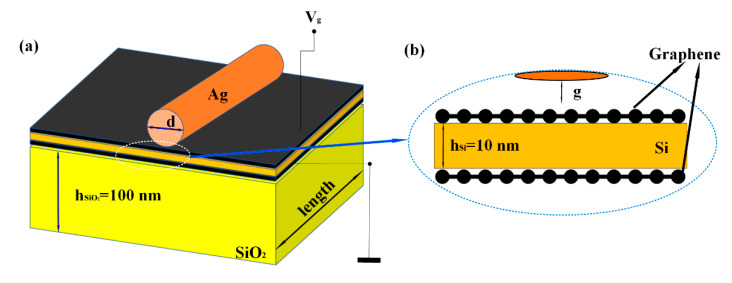
The stereograph and sectional view of graphene electro-optical switch modulator based on silver nanowire: (**a**) 3D layout structure (**b**) cross-sectional structure.

Figure 1 shows the three-dimensional and sectional model of the structure. The modulator is composed of a hybrid waveguide structure, which is constructed of an SiO_2_ substrate, graphene–Si–graphene heterostructure and Ag nanowire. Furthermore, the graphene in this modulator is above and below the Si. Moreover, the whole structure is surrounded by SiO_2_ cladding, which include the gap between upper graphene and Ag.

In this structure, h_SiO2_ denotes the height of SiO_2_ substrate and its value is 100 nm. R denotes the radius of Ag nanowire and the range of R is from 10 nm to 100 nm. The thickness of single-layer graphene is 0.7 nm and h_Si_ denotes the height of Si (h_Si_ = 10 nm). Furthermore, g denotes the gap between Ag nanowire and graphene, whose range is from 1 nm to 10 nm. Using the relationship between voltages and propagation length of this switch modulator, its length can be obtained and achieve about 100% modulation depth, which will be discussed in detail in Figures 6 and 7.

For the good optical properties of graphene, its Fermi level can be controlled by external voltages [36]. The relationship between carrier density and Fermi level is calculated by E_F_ = ħV_f_(π·n_0_)^1/2^, where ħ is the reduced Planck constant. V_f_ denotes the Fermi velocity. For graphene, its Fermi velocity is 1.1 × 10^6^ m/s. By definition, n_0_ is the carrier concentration and its value can be calculated by n_0_ = ε_0_ε_r_(U + U_0_)/(d·e). U is the voltage applied to graphene. U_0_ is related to the Fermi level of graphene when no voltage is applied, and its value is usually taken as 0 V. ε_0_ refers to the dielectric constant in vacuum, and the general value is about 8.854 × 10^−12^ F/m. ε_r_ denotes the dielectric constant of substrate materials and e is electron charge; d presents the thickness of substrates, and whose value is equal to h_is_. 

**Figure 2:**
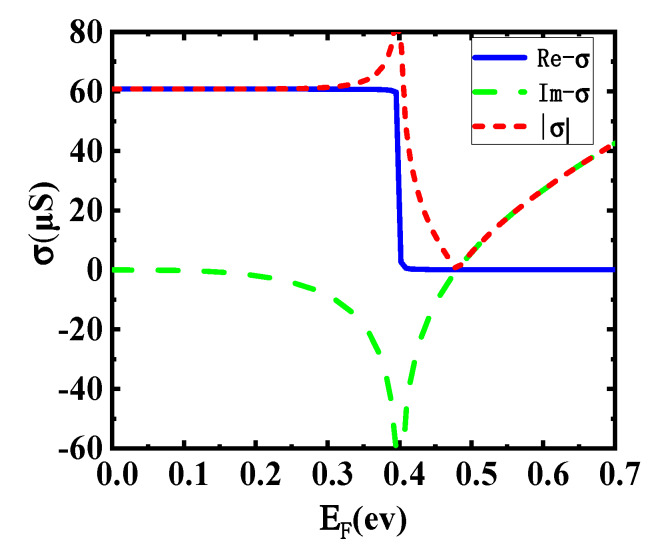
Dependence of graphene conductivity on the Fermi level in 1550 nm incident wavelength.

At temperature T = 296 K, the relationship between the Fermi level and electrical conductivity of graphene is given by Equation (1) [31].

$$\sigma_{intra}=\sigma_{0}\frac{4E_{F}}{\pi }\frac{1}{\hbar \tau_{1}-i\hbar \omega }$$

$$\sigma_{\mathrm{int}\mathrm{er}}^{\prime }=\sigma_{0}(1+\frac{1}{\pi }\arctan\frac{\hbar \omega -E_{F}}{\hbar \tau_{2}}-\frac{1}{\pi }\arctan\frac{\hbar \omega +2E_{F}}{\hbar \tau_{2}})$$

$$\sigma_{\mathrm{int}er}^{\prime \prime }=-\sigma_{0}\frac{1}{2\pi }\ln\frac{{\left(2E_{F}+\hbar \omega \right)}^{2}+\hbar^{2}\tau_{2}^{2}}{{\left(2E_{F}-\hbar \omega \right)}^{2}+\hbar^{2}\tau_{2}^{2}}$$

(1) $$\sigma =\sigma_{\mathrm{int}ra}+\sigma_{\mathrm{int}er}^{\prime }+i\sigma_{\mathrm{int}er}^{\prime \prime }$$

where *σ* is the conductivity of monolayer graphene. *σ* is consisted by intraband conductivity *σ_intra_* and interband conductivity *σ_inter_*. *σ_intra_* denotes to the electrical conductivity generated by in-band free carrier transitions. *σ_inter_* refers to the electrical conductivity generated by free carrier transition between the bands. ω is the angular frequency of incident light and the incident wavelength is 1550 nm at here. τ_1_ and τ_2_ are the intraband and interband relaxation time of graphene, and the values are 10 fs and 1.2 ps, respectively [31,37]. In addition, σ_0_ is equal to πe^2^/2ħ. Figure 2 shows the image of conductivity as a function of the Fermi level in 1550 nm incident wavelength. 

**Figure 3:**
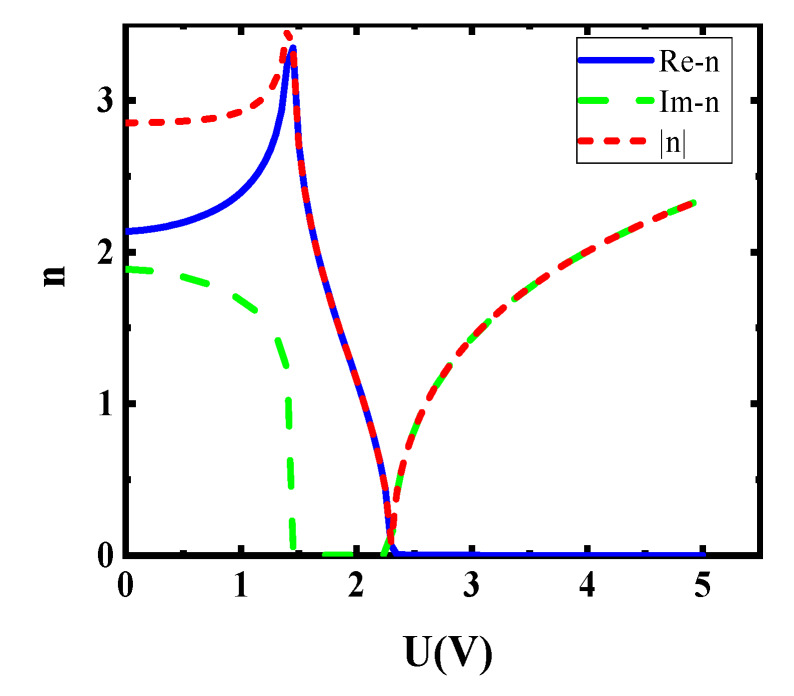
Dependence of graphene refractive index on the external voltage in 1550 nm incident wavelength.

The relationship between conductivity and permittivity can be given by Equation (2):

(2) $$\varepsilon (\omega )=1+\frac{i\sigma (w)}{\omega \varepsilon_{0}d_{G}}$$

where d_G_ denotes the thickness of single-layer graphene. According to Equation (2), the real part of the dielectric constant decides the imaginary part of the conductivity, while the imaginary part of the dielectric constant decides the real part of conductivity. The refractive index can be given by n = (*ε*(ω))^1/2^. According to this way, the relationship about voltage and the refractive index of graphene can be shown in Figure 3. 

The propagation length reflects the device’s transmission properties, which is the length that the electric field decays to 1/e of itself. Larger propagation length means the device has better transmission ability. It is generally believed that the electric field intensity beyond the propagation length cannot be detected. The propagation length is calculated by L_m_ = λ/[4πIm(N_eff_)] [27,38], where λ denotes the incident wavelength and N_eff_ refers to the effective refractive index of the modulator.

The normalized effective mode area A_eff_/A_0_ can be given by Equation (3) [38]:

$$A_{0}=\lambda^{2}/4$$

(3) $$A_{eff}=\frac{W_{m}}{\max\{W(x,y))}=\frac{\int \int_{-\infty }^{+\infty }W(x,y)dxdy}{\max\{W(x,y))}$$

where W_m_ refers to the total energy density and max {W(x,y)} represents the maximum energy density of the structure. Smaller A_eff_/A_0_ means stronger optical confinement in the devices. The consumed energy can be given by E = 1/4CV_p_^2^. C represents the total capacitance of the modulator, which is mainly composed of the quantum capacitance of graphene and the equivalent capacitance between graphene and Si. V_p_ refers to the peak-to-peak value of external voltage. The 3dB bandwidth of the modulator can be given by f_3dB_ = 1/2πτ_RC_ = 1/2πR_total_C, where R_total_ refers to the resistance of the whole modulator. It is composed of the graphene resistance and the contact resistance between graphene and metal electrode. The resistance of graphene can be generally ignored because of the small value (about tens of ohms) [39].

The modal properties of the presented modulator are investigated numerically by using the finite element method package in RF module of COMSOL Multiphysics 5.4 software [40]. The mode analysis with scattering boundary is used to simulate the open boundary condition and to calculate relative propagation length and the normalized effective mode area of the structure. In this simulation, the all device size is set 400 nm × 400 nm in x and y direction. The incident wavelength λ is 1550 nm. The relative permittivities of Ag, Si and SiO_2_ at 1550 nm incident wavelength are − 129 + 3.3i, 2.25 and 12.25, respectively [27].

## 3. Results and Discussion

In order to optimize the size of modulator, the normalized effective mode area and propagation length need to be calculated. By discussing the values of the normalized effective mode area and propagation, the optimal size of gap g and radius R can be decided.

**Figure 4:**
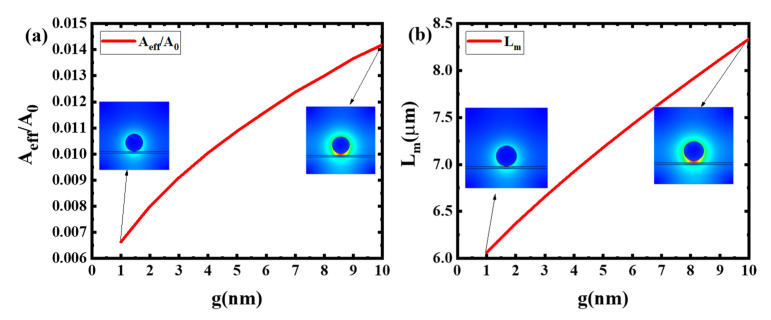
Dependence of normalized effective mode area and propagation length on the gap g at R = 50 nm: (**a**) A_eff_/A_0_ (**b**) L_m_.

**Figure 5:**
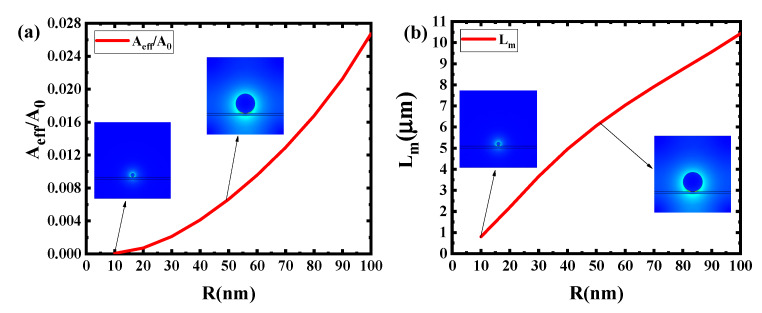
Dependence of normalized effective mode area and propagation length on the radius R at g = 1 nm: (**a**) A_eff/_A_0_ (**b**) L_m_.

It can be concluded from Figure 4 that the change trend of A_eff_/A_0_ becomes larger with the increase of g. The main reason is that when g is large, the interface area between dielectric Si and Ag nanowire is not large enough to confine the light into a very small scale. The L_m_ becomes larger with the increase of g. The main reason is that the increase of g can bring the smaller interface area between dielectric Si and Ag nanowire, which will decrease the energy loss of incident light and bring the increase of L_m_. Figure 5 shows that L_m_ will increase with the larger R. The key factor is that the larger radius will cause a smaller loss of incident light and improve the propagation length. By contrast, larger R reduces the degree of light localization and results in a larger A_eff_/A_0_. In fact, the results shows that larger L_m_ and smaller A_eff_/A_0_ are contradictory in the devices. In order to obtain the larger L_m_ and keep A_eff_/A_0_ as small as possible, the g is set to 1 nm and R is set to 50 nm. By tuning the refractive index of graphene, N_eff_ will be changed and the propagation length can be adjusted for this switch modulator.

**Figure 6:**
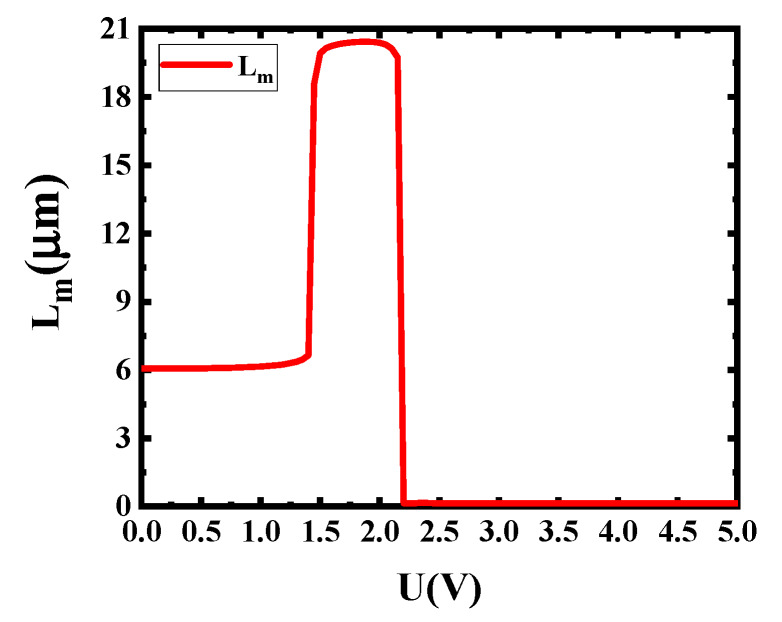
Dependence of L_m_ on external voltage for the proposed modulator graphene electro-optical switch modulator based on silver nanowire (GESMBOSN).

**Figure 7:**
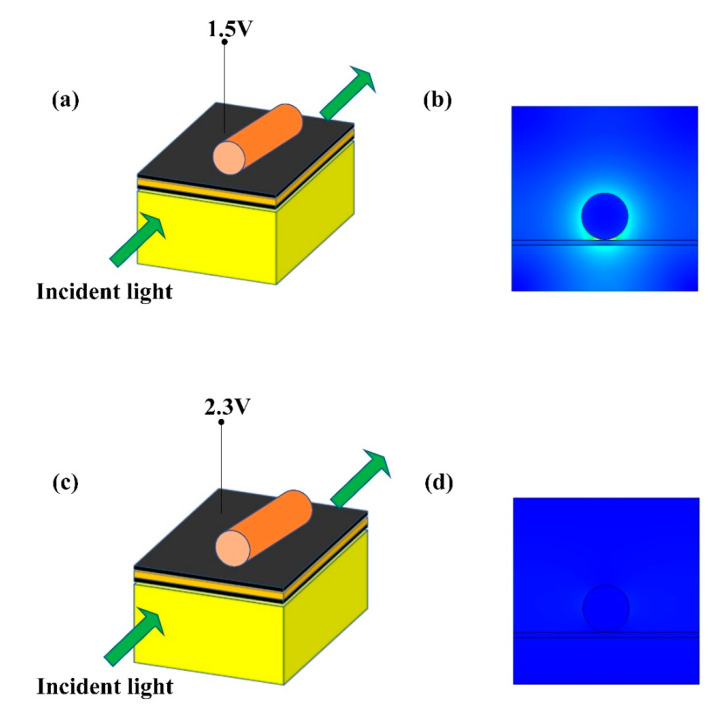
Working principle diagram of the presented modulator GESMBOSN: (**a**) 3D layout structure in 1.5 V (**b**) two-dimensional electric field pattern after modulation in 1.5 V. (**c**) 3D layout structure in 2.3 V (**d**) two-dimensional electric field pattern after modulation in 2.3 V.

**Figure 8:**
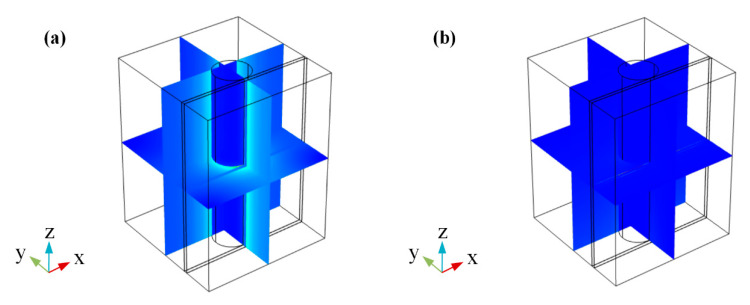
Multi section graph of GESMBOSN’s three-dimensional electric field in different external voltages: (**a**) 1.5 V (**b**) 2.3 V.

**Figure 9:**
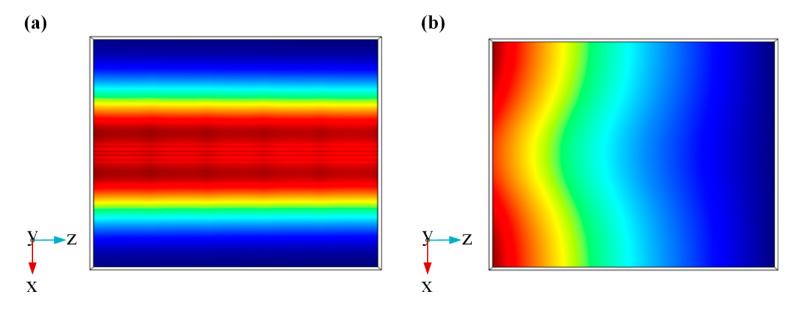
Electric field transmission cross section of GESMBOSN in different external voltages: (**a**) 1.5 V (**b**) 2.3 V.

**Figure 10:**
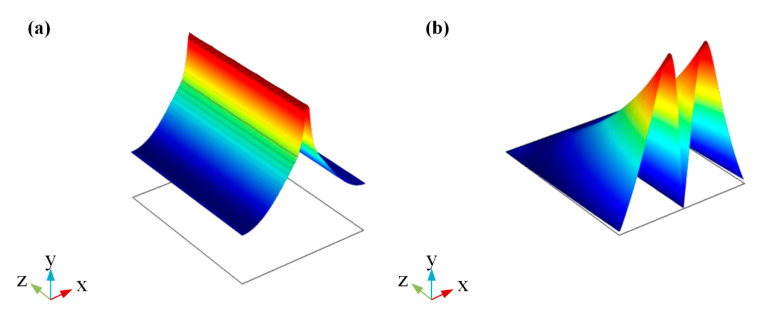
Electric field intensity distribution diagram of GESMBOSN in different external voltages: (**a**) 1.5 V (**b**) 2.3 V.

According to the result in Figure 6, the 1.5 V (corresponding to 19.93 μm propagation length) is used as the light passing voltage and the 2.3 V (corresponding to 0.14 μm propagation length) is used as the light cut-off voltage. Considering the factor for keeping device sizes as small as possible and the results in Figure 6, the modulator length is set to 3 μm and about 100% modulation depth can be obtained. In this case, the modulation function of this switch modulator can be achieved. Figure 7 shows the working principle of this switch modulator. When different voltages are applied to this switch modulator, different electric field distribution is shown in Figure 7b,d. The high electric field intensity in Figure 7b illustrates that the incident light can pass this modulator. By contrast, the low electric field intensity in Figure 7d illustrates that the incident light cannot pass this modulator. With this design method, voltages are applied to both ends of graphene, which can control the light absorption by tuning graphene, so as to realize the change of propagation length for an electro-optical modulator. As shown in Figure 8, Figure 9 and Figure 10, the incident light will pass this switch modulator at 1.5 V. By contrast, the incident light will disappear in this switch modulator at 2.3 V. In this way, the function of signal modulation can be realized.

The total capacitance is mainly composed of graphene quantum capacitance and equivalent capacitance between graphene and Si. After calculation, the quantum capacitance of graphene is ~0.098 F/m and the equivalent capacitance between graphene and Si is ~0.0108 F/m. Due to the width and length of this switch modulator is 400 nm and 3 μm, its total capacitance is 10.66 fF. Moreover, 1.5 V and 2.3 V voltages are used to control the on and off for this switch modulator. According to Equation E = 1/4CV_p_^2^, the energy loss of the whole switch modulator is ~1.71 fJ/bit. The total resistance mainly includes graphene resistance and the contact resistance between graphene and metal electrode. Because the resistance of graphene is too small (about tens of ohms) and can be ignored, the total resistance is 177.93 ohms. According to Equation f_3dB_ = 1/2πτ_RC_ = 1/2πR_total_C, the 3 dB bandwidth of this switch modulator is 83.91 GHz.

## 4. Fabrication Error Tolerance and Manufacturing Process

**Figure 11:**
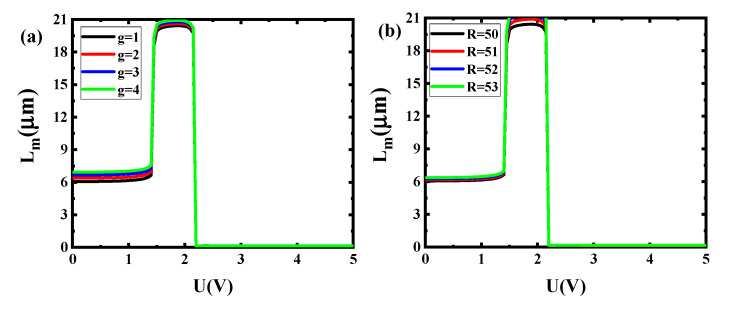
Dependence of L_m_ on external voltage in different fabrication error tolerance for the proposed modulator GESMBOSN: (**a**) g (**b**) R.

It can be observed from Figure 11a that the change of g only has a little influence on the propagation length. The maximum error of propagation length is given by (max(L_m_) − min(L_m_))/max(L_m_). The maximum error of this switch modulator in 1.5 V is about 2.89% (g = 1 corresponds to the ~19.926 μm propagation length and g = 4 corresponds the ~20.502 μm propagation length). For the error of propagation length in 2.3 V, maximum error of this switch modulator is about 0.58% (g = 1 corresponds to the ~0.1396 μm propagation length and g = 4 corresponds to the ~0.1404 μm propagation length). As shown in Figure 11b, the maximum error of this switch modulator in 1.5 V is about 8.84% (R = 50 corresponds the ~19.926 μm propagation length and R = 54 corresponds to the ~21.688 μm propagation length). For the error of propagation length in 2.3 V, maximum error of this switch modulator is about 5.74% (R = 50 corresponds the ~0.1396 μm propagation length and R = 54 corresponds to the ~0.1477 μm propagation length). The results prove that this switch modulator has excellent robustness.

The switch modulator can be manufactured with general commercial SOI wafers. Firstly, two grating couplers can be produced at both ends of the device by deep reactive ion etching (The cycle of grating couplers is set to 780 nm in 1550 nm working length), which can achieve the phase matching and let the incident light enter or output the switch modulator. Then a layer of graphene can be transferred onto 100 nm SiO_2_. 10 nm thickness of Si upon the graphene layer is deposited as the substrate by atomic layer deposition, which can constrain the electric field distribution inside the device. Another layer of graphene can be transferred in the same way. Then, a SiO_2_ layer can be transferred onto graphene, which can be used as a cladding material for the gap between Ag and the upper graphene. In addition, the SiO_2_ layer is also used to grow a silver nanowire. Flipping the entire structure, by ion beam etching an SiO_2_ layer in a z direction definite size, a cylindrical hole can be etched out. After using the magnetron sputtering technology and masks technology, silver ions can fill empty slots in the cylinder to form the silver nanowire needed. Finally, flipping the entire structure to the original place, SiO_2_ cladding can be grown on the whole devices [41,42]. In this way, an Ag nanowire can be put on the single sheet of graphene and we can precisely control the gap between silver and graphene.

## 5. Conclusions

In this paper, a novel graphene electro-optical switch modulator based on adjusting propagation length is proposed in the near infrared band. It successfully combines the advantages of hybrid waveguide and the tunability of graphene to adjust the propagation length, which achieves ~100% modulation depth for the switch modulator in theory. The numerical simulations at the wavelength of 1550 nm show that GESMBOSN has relatively low loss (~1.71 fJ/bit) and very large 3 dB bandwidth (~83.91 GHz). In addition, the wonderful robustness of this switch modulator is also conducive to the actual application. Most importantly, with this new method to design a graphene switch modulator, other graphene switch modulators can also have the potential to arrive at about 100% modulation depth.

## References

[1] Novoselov K., Geim A.K., Morozov S., Jiang D., Zhang Y., Dubonos S.V., Grigorieva I.V., Firsov A.A. (2004). Electric Field Effect in Atomically Thin Carbon Films. Science.

[2] Lee Y., Yu S.H., Jeon J., Kim H., Lee J.-Y., Kim H., Ahn J.-H., Hwang E., Cho J.H. (2015). Hybrid Structures of Organic Dye and Graphene for Ultrahigh Gain Photodetectors. Carbon.

[3] Gan X., Shiue R.-J., Gao Y., Meric I., Heinz T.F., Shepard K., Hone J., Assefa S., Englund D. (2013). Chip-Integrated Ultrafast Graphene Photodetector with High Responsivity. Nat. Photon..

[4] Li J., Tao J., Chen Z.H., Huang X.-G. (2016). All-optical Controlling Based on Nonlinear Graphene Plasmonic Waveguides. Opt. Express.

[5] Li H., Anugrah Y., Koester S.J., Li M. (2012). Optical absorption in graphene integrated on silicon waveguides. Appl. Phys. Lett..

[6] Sorianello V., Midrio M., Contestabile G., Asselberghs I., Van Campenhout J., Huyghebaert C., Goykhman I., Ott A.K., Ferrari A.C., Romagnoli M. (2017). Graphene–Silicon Phase Modulators with Gigahertz Bandwidth. Nat. Photon..

[7] Chen J.-H., Zheng B.-C., Shao G.-H., Ge S.-J., Xu F., Lu Y.-Q. (2015). An All-Optical Modulator Based on a Stereo Graphene–Microfiber Structure. Light. Sci. Appl..

[8] Cao H., Zhou X., Qin Z., Liu Z. (2013). Low-Temperature Preparation of Nitrogen-Doped Graphene for Supercapacitors. Carbon.

[9] Zhu Y., Murali S., Stoller M.D., Ganesh K.J., Cai W., Ferreira P.J., Pirkle A., Wallace R.M., Cychosz K.A., Thommes M. (2011). Carbon-Based Supercapacitors Produced by Activation of Graphene. Science.

[10] Wan S., Bi H., Zhou Y., Xie X., Su S., Yin K., Sun L. (2017). Graphene Oxide as High-Performance Dielectric Materials for Capacitive Pressure Sensors. Carbon.

[11] Zhang X., Dai Z., Si S., Wu W., Deng H., Wang F., Xiao X., Jiang C. (2017). Mercuric Contamination: Ultrasensitive SERS Substrate Integrated with Uniform Subnanometer Scale “Hot Spots” Created by a Graphene Spacer for the Detection of Mercury Ions (Small 9/2017). Small.

[12] Simsek E. (2013). A Closed-Form Approximate Expression for the Optical Conductivity of Graphene. Opt. Lett..

[13] Gusynin V.P., Sharapov S., Carbotte J.P. (2006). Magneto-Optical Conductivity in Graphene. J. Phys. Condens. Matter.

[14] Zhang J., Ouyang H., Zheng X., You J., Chen R., Zhou T., Sui Y., Liu Y., Cheng X.A., Jiang T. (2018). Ultrafast Saturable Absorption of MoS_2 Nanosheets under Different Pulse-Width Excitation Conditions. Opt. Lett..

[15] Dalir H., Xia Y., Wang Y., Zhang X. (2016). Athermal Broadband Graphene Optical Modulator with 35 GHz Speed. ACS Photon..

[16] Youngblood N., Anugrah Y., Ma R., Koester S.J., Li M. (2014). Multifunctional Graphene Optical Modulator and Photodetector Integrated on Silicon Waveguides. Nano Lett..

[17] Lin H., Song Y., Huang Y., Kita D., Deckoff-Jones S., Wang K., Li L., Li J., Zheng H., Luo Z. (2017). Chalcogenide Glass-on-Graphene Photonics. Nat. Photon..

[18] Yan J., Ma C., Huang Y., Yang G., Huang Y. (2019). Single Silicon Nanostripe Gated Suspended Monolayer and Bilayer WS2 to Realize Abnormal Electro-Optical Modulation. Mater. Horizons.

[19] Phare C., Lee Y.-H.D., Cardenas J., Lipson M. (2015). Graphene Electro-Optic Modulator with 30 GHz Bandwidth. Nat. Photon..

[20] Liu M., Yin X., Ulin-Avila E., Geng B., Zentgraf T., Ju L., Wang F., Zhang X. (2011). A Graphene-Based Broadband Optical Modulator. Nature.

[21] Nielsen M.P.P., LaFone L., Rakovich Y.P., Sidiropoulos T., Rahmani M., Maier S.A., Oulton R.F. (2016). Adiabatic Nanofocusing in Hybrid Gap Plasmon Waveguides on the Silicon-on-Insulator Platform. Nano Lett..

[22] Dabos G., Manolis A., Papaioannou S., Tsiokos D., Markey L., Weeber J.-C., Dereux A., Giesecke A.L., Porschatis C., Chmielak B. (2018). CMOS Plasmonics in WDM Data Transmission: 200 Gb/s (8 × 25Gb/s) Transmission over Aluminum Plasmonic Waveguides. Opt. Express.

[23] Veronis G., Fan S. (2005). Bends and Splitters in Metal-Dielectric-Metal Subwavelength Plasmonic Waveguides. Appl. Phys. Lett..

[24] Butt M.A., Хoнина C.H., Kazanskiy N.L. (2019). Plasmonic Refractive Index Sensor Based on Metal-Insulator-Metal Waveguides with High Sensitivity. J. Mod. Opt..

[25] Oulton R.F., Sorger V.J., Genov D.A., Pile D., Zhang X. (2008). A Hybrid Plasmonic Waveguide for Subwavelength Confinement and Long-Range Propagation. Nat. Photon..

[26] Bian Y., Ren Q., Kang L., Yue T., Werner P.L., Werner D.H. (2017). Deep-Subwavelength Light Transmission in Hybrid Nanowire-Loaded Silicon Nano-Rib Waveguides. Photon. Res..

[27] Dong L., Liu H., Wang S., Qu S., Wu L. (2017). Hybrid Tube-Triangle Plasmonic Waveguide for Ultra-Deep Subwavelength Confinement. J. Light. Technol..

[28] Bian Y., Gong Q. (2014). Deep-Subwavelength Light Confinement and Transport in Hybrid Dielectric-Loaded Metal Wedges. Laser Photon. Rev..

[29] Butt M.A., Khonina S.N., Kazanskiy N.L. (2018). Hybrid Plasmonic Waveguide-Assisted Metal–Insulator–Metal Ring Resonator for Refractive Index Sensing. J. Mod. Opt..

[30] Wang Y., Liu H., Wang S., Cai M., Zhang H., Qiao Y. (2020). Electrical Phase Control Based on Graphene Surface Plasmon Polaritons in Mid-infrared. Nanomaterials.

[31] Qu S., Ma C., Liu H. (2017). Tunable Graphene-Based Hybrid Plasmonic Modulators for Subwavelength Confinement. Sci. Rep..

[32] Ansell D., Radko I.P., Han Z., Rodriguez F., Bozhevolnyi S.I., Grigorenko A.N. (2015). Hybrid Graphene Plasmonic Waveguide Modulators. Nat. Commun..

[33] Ghosh R.R., Bhardwaj P., Subramanian S., Jaiswal M., Dhawan A. (2019). Design of Electro-Optic Modulators and Switches Based on Graphene and Phase Change Materials. Integr. Opt. Des. Devices Syst. Appl. V.

[34] Zheng P., Yang H., Fan M., Hu G., Zhang R., Yun B., Cui Y. (2018). A Hybrid Plasmonic Modulator Based on Graphene on Channel Plasmonic Polariton Waveguide. Plasmonics.

[35] Chen X., Wang Y., Xiang Y., Jiang G., Wang L., Bao Q., Zhang H., Liu Y., Wen S., Fan D. (2016). A Broadband Optical Modulator Based on a Graphene Hybrid Plasmonic Waveguide. J. Light. Technol..

[36] Wang Y., Liu H., Wang S., Cai M., Ma L. (2019). Optical Transport Properties of Graphene Surface Plasmon Polaritons in Mid-Infrared Band. Crystals.

[37] Cai M., Wang S., Gao B., Wang Y., Han T., Liu H. (2018). A New Electro-Optical Switch Modulator Based on the Surface Plasmon Polaritons of Graphene in Mid-Infrared Band. Sensors.

[38] Ma Y., Farrell G., Semenova Y., Wu Q. (2015). A Hybrid Wedge-To-Wedge Plasmonic Waveguide with Low Loss Propagation and Ultra-Deep-Nanoscale Mode Confinement. J. Light. Technol..

[39] Qu S., Ma C., Wang S., Liu H., Dong L. (2018). Modulation Speed Limits of a Graphene-Based Modulator. Opt. Quantum Electron..

[40] Gric T., Cada M. (2014). Analytic Solution to Field Distribution in One-Dimensional Inhomogeneous Media. Opt. Commun..

[41] Wang Y., Wang S., Cai M., Liu H. (2019). A Long Propagation Distance Hybrid Triangular Prism Waveguide for Ultradeep Subwavelength Confinement. IEEE Sens. J..

[42] Bian Y., Zheng Z., Liu Y., Liu J., Zhu J., Zhou T. (2011). Hybrid Wedge Plasmon Polariton Waveguide with Good Fabrication-Error-Tolerance for Ultra-Deep-Subwavelength Mode Confinement. Opt. Express.

